# Associations of inter-annual rainfall decreases with subsequent HIV outcomes for persons with HIV on antiretroviral therapy in Southern Africa: a collaborative analysis of cohort studies

**DOI:** 10.1186/s12879-023-08902-9

**Published:** 2023-12-19

**Authors:** Adam Trickey, Leigh F. Johnson, Fai Fung, Rogerio Bonifacio, Collins Iwuji, Samuel Biraro, Samuel Bosomprah, Linda Chirimuta, Jonathan Euvrard, Geoffrey Fatti, Matthew P. Fox, Per Von Groote, Joe Gumulira, Guy Howard, Lauren Jennings, Agnes Kiragga, Guy Muula, Frank Tanser, Thorsten Wagener, Andrea Low, Peter Vickerman

**Affiliations:** 1 Population Health Sciences, University of Bristol, Bristol, UK; 2 Centre for Infectious Disease Epidemiology and Research, School of Public Health and Family Medicine, University of Cape Town, Cape Town, South Africa; 3 Department of Civil Engineering, University of Bristol, Bristol, UK; 4 UK Meteorological Office, Exeter, UK; 5 Climate and Earth Observation Unit, Research Assessment and Monitoring Division, World Food Programme HQ, Rome, Italy; 6 Africa Health Research Institute, KwaZulu-Natal, South Africa; 7 Department of Global Health Infection, Brighton and Sussex Medical School, University of Sussex, Brighton, UK; 8 ICAP at Columbia University, Nakasero, Kampala, Uganda; 9 Centre for Infectious Disease Research in Zambia, Lusaka, Zambia; 10 Department of Biostatistics, School of Public Health, University of Ghana, Legon, Accra, Ghana; 11 Newlands Clinic, Harare, Zimbabwe; 12 Kheth’Impilo AIDS Free Living, Cape Town, South Africa; 13 Division of Epidemiology and Biostatistics, Department of Global Health, Faculty of Medicine and Health Sciences, Stellenbosch University, Cape Town, South Africa; 14 Health Economics and Epidemiology Research Office, Faculty of Health Sciences, University of the Witwatersrand, Johannesburg, South Africa; 15 Department of Global Health and Department of Epidemiology, Boston University School of Public Health, Boston, MA USA; 16 Institute of Social and Preventive Medicine, University of Bern, Bern, Switzerland; 17 Lighthouse Trust, Mzimba, Malawi; 18 Department of Civil Engineering and Cabot Institute of the Environment, University of Bristol, Bristol, UK; 19 Desmond Tutu Health Foundation, Institute of Infectious Diseases and Molecular Medicine, Department of Medicine, University of Cape Town, Cape Town, South Africa; 20 Research Division, African Population and Health Research Center, Nairobi, Kenya; 21 Centre for Epidemic Response and Innovation, School of Data Science and Computational Thinking, Stellenbosch University, Stellenbosch, South Africa; 22 School of Nursing and Public Health, University of KwaZulu-Natal, Durban, South Africa; 23 Institute of Environmental Science and Geography, University of Potsdam, Potsdam, Germany; 24 Department of Epidemiology, Mailman School of Public Health, Columbia University, New York, NY USA; 25 NIHR Health Protection Research Unit in Behavioural Science and Evaluation at University of Bristol, Bristol, UK

**Keywords:** ARV, Treatment, PLHIV, Climate change, Drought

## Abstract

**Background:**

Periods of droughts can lead to decreased food security, and altered behaviours, potentially affecting outcomes on antiretroviral therapy (ART) among persons with HIV (PWH). We investigated whether decreased rainfall is associated with adverse outcomes among PWH on ART in Southern Africa.

**Methods:**

Data were combined from 11 clinical cohorts of PWH in Lesotho, Malawi, Mozambique, South Africa, Zambia, and Zimbabwe, participating in the International epidemiology Databases to Evaluate AIDS Southern Africa (IeDEA-SA) collaboration. Adult PWH who had started ART prior to 01/06/2016 and were in follow-up in the year prior to 01/06/2016 were included. Two-year rainfall from June 2014 to May 2016 at the location of each HIV centre was summed and ranked against historical 2-year rainfall amounts (1981–2016) to give an empirical relative percentile rainfall estimate. The IeDEA-SA and rainfall data were combined using each HIV centre’s latitude/longitude. In individual-level analyses, multivariable Cox or generalized estimating equation regression models (GEEs) assessed associations between decreased rainfall versus historical levels and four separate outcomes (mortality, CD4 counts < 200 cells/mm^3^, viral loads > 400 copies/mL, and > 12-month gaps in follow-up) in the two years following the rainfall period. GEEs were used to investigate the association between relative rainfall and monthly numbers of unique visitors per HIV centre.

**Results:**

Among 270,708 PWH across 386 HIV centres (67% female, median age 39 [IQR: 32–46]), lower rainfall than usual was associated with higher mortality (adjusted Hazard Ratio: 1.18 [95%CI: 1.07–1.32] per 10 percentile rainfall rank decrease) and unsuppressed viral loads (adjusted Odds Ratio: 1.05 [1.01–1.09]). Levels of rainfall were not strongly associated with CD4 counts < 200 cell/mm^3^ or > 12-month gaps in care. HIV centres in areas with less rainfall than usual had lower numbers of PWH visiting them (adjusted Rate Ratio: 0.80 [0.66–0.98] per 10 percentile rainfall rank decrease).

**Conclusions:**

Decreased rainfall could negatively impact on HIV treatment behaviours and outcomes. Further research is needed to explore the reasons for these effects. Interventions to mitigate the health impact of severe weather events are required.

**Supplementary Information:**

The online version contains supplementary material available at 10.1186/s12879-023-08902-9.

## Introduction

Climate change is increasing the frequency and severity of extreme weather events, including heatwaves, flooding, cyclones, and meteorological droughts [1], defined as an exceptional lack of rainfall compared to normal circumstances [2]. These severe weather anomalies impact human health through various mechanisms, including altered patterns of vector-borne and water-borne diseases linked to drought [3], and heat-related illnesses [4]. Southern Africa is one of the regions most affected by climate change [1] due to increasing risks of drought caused by changes in precipitation and limited water storage, as well as limited capacity and resources to support adaptation [1]. Drought is an ongoing and worsening trend [5]. The fraction of sub-Saharan Africa experiencing severe drought increased from < 5% in 1901 to ~ 15% in 2013 [6], and is projected to further increase in this century [7].

Climate change has been hypothesised by UNAIDS to impact the HIV epidemic in settings where HIV prevalence is highest [8], such as Southern Africa [9]. A lack of rainfall impacts vegetation, including agricultural production [10]. This can lead to increased food insecurity and poverty, which in turn can lead to behaviours that result in sub-optimal HIV treatment outcomes through a variety of mechanisms [11, 12]. For instance, evidence suggests that the resulting income shocks caused by drought and food insecurity can disrupt antiretroviral therapy (ART) treatment schedules [13]. This may occur through reduced adherence [14] due to people finding it difficult to travel to clinics because they have to prioritise food and money over attending clinics for ART [13], potentially leading to decreases in CD4 cell counts [15] and increases in viral load [16]. Additionally, some ART medications are more effective when taken with food [17], while some can be taken with or without food. Unfortunately, research into this field has so far been limited because of a lack of cross-disciplinary expertise, difficulties in identifying appropriate data, and complexity in the causal pathway between climate exposures and HIV-related outcomes.

Using a large, established multi-country HIV clinical cohort in Southern Africa, linked with spatial and temporal data on levels of rainfall, we investigated whether living somewhere that has recently had levels of rainfall lower than historical averages is associated with subsequent adverse treatment outcomes among persons with HIV (PWH) on ART.

## Methods

### Cohort data

The International epidemiology Databases to Evaluate AIDS (IeDEA) (https://www.iedea.org/) is an international research consortium that collects deidentified patient-level data from approximately two million PWH across 46 countries [18]. IeDEA Southern Africa [IeDEA-SA] (https://www.iedea.org/regions/southern-africa/), one of four African IeDEA regions, comprises ART programs that collect data from facilities across Lesotho, Malawi, Mozambique, South Africa, Zambia and Zimbabwe [19]. Local review boards and ethics committees approved the use of IeDEA data for research within the IeDEA collaboration. The Ethics Committee of the Canton of Bern (150/14, PB 2016–00273), Switzerland, approved data merging and collaborative analyses of the IeDEA-SA data (the methods of individual IeDEA-SA concept sheets are not reviewed by an ethics committee). Informed consent for the use of IeDEA routinely collected data has been obtained or waived according to local requirements of each cohort. For this analysis, data were available for 11 IeDEA-SA HIV cohorts from all included countries that had longitude and latitude coordinates available for their participating HIV centres.

### Rainfall data

We used longitudinal, gridded rainfall data to develop a measure to capture weather shocks compared with a historical norm for each location in our study. Data on rainfall estimates from the Climate Hazards Group InfraRed Precipitation with Station Data (CHIRPS) at 0.05° resolution (roughly 30km^2^) were used to quantify rainfall [20]. This gridded dataset was prepared by the Vulnerability Analysis and Mapping (VAM) Geospatial Analysis Team at the Analysis and Trends Service of the World Food Programme (WFP). The 2-year total rainfall from June 2014 to May 2016 for each grid location was summed and then ranked compared to all 2-year rainfall amounts within the 1981–2016 period for that grid location and converted to an empirical relative percentile rank. This produced a variable with values from 1–100, with 50 indicating median rainfall versus historical levels, and lower values indicating less rainfall than usual [21]. We combined the IeDEA-SA HIV cohort data with the gridded data on rainfall using latitude and longitude data of each HIV centre and overlaying them with the gridded rainfall dataset. Further information on the climate contexts in the region during this time-period are given in the supplementary materials. The 2014–2016 period was chosen as Southern Africa experienced a severe drought during this time-period [21].

### Individual-level inclusion criteria

PWH on ART were eligible for inclusion if they had started ART prior to 1st June 2016, were aged ≥ 16 years on this date, had a recorded follow-up visit between 1st June 2015 and the 31st May 2016, and had not been recorded by the cohorts as lost-to-follow-up or dead before the 1st June 2016. PWH were dropped from the analyses if they were missing data on which HIV centre they had attended (*N* = 43,531). A small number of PWH were dropped who attended HIV centres that did not have longitude or latitude coordinates available (*N* = 169), were recorded as having closed prior to 2016 (*N* = 32), had missing data on age (*N* = 28) or sex at birth (*N* = 24), or started ART before 2000 (*N* = 11). For each separate analysis, further cohort-level exclusions were made to ensure the included cohorts captured the outcomes of interest. For the analysis of all-cause mortality, one cohort (CIDRZ) was dropped due to suspected under-ascertainment of mortality, determined by a very low percentage of deaths compared to the other cohorts, whilst another (Kheth’Impilo) was dropped due to a lack of data on dates of death. For the analyses of CD4 counts < 200 cells/mm^3^ or viral load > 400 copies/mL, cohorts were dropped if > 50% of eligible PWH were missing data on the respective outcomes during follow-up. Additionally, for these two analyses, cohorts were dropped if > 50% of eligible PWH were missing a CD4 count or viral load measurement, respectively, at follow-up start. Eight cohorts (Lighthouse Trust, SMART Zimbabwe, Hlabisa, Gugulethu Community Health Centre, Themba Lethu, Khayelitsha, CIDRZ, and SMART Lesotho) were dropped from the CD4 analysis and five were dropped from the viral load analysis (CIDRZ, SMART Lesotho, SMART Mozambique, SMART Zimbabwe, and Lighthouse Trust).

### Individual-level analyses

Our analyses investigated whether changes in rainfall between June 2014 – May 2016 (compared with historical values) affected HIV outcomes up to 2 years afterwards. Four outcomes were investigated in individual-level analyses. For each outcome of interest, models adjusted for cohort and evaluated the association of the outcome per 10 percentile relative decreases in rainfall rank (the scale of the rainfall variable was reversed for interpretability). Although we considered using quadratic terms to account for both rainfall extremes (drought and flooding) only one centre in the dataset experienced a relative rainfall value that could be considered extremely high as compared to historical values (≥ 85), so we instead included just a linear term.

The associations between the outcomes of all-cause mortality and gaps in follow-up of ≥ 12 months with the variable on the relative change in rainfall were analysed using Cox proportional hazards models. For analyses where CD4 counts < 200 cells/mm^3^ or viral load > 400 copies/mL were the (binary) outcomes, generalized estimating equation (GEE) regression models were fitted (binomial, with a logit link) accounting for each recorded CD4 or viral load value (up to the 1st June 2018) and additionally adjusting for the time between follow-up start and the date of each recorded value.

Follow-up started on 1st June 2016 and ended at the earliest of either the cohort/centre-specific administrative censoring date, 1st June 2018, or, for the analyses of all-cause mortality and gaps in follow-up of > 1 year, the date of this outcome. The end date 1st June 2018 was chosen to limit the period in which the outcomes could be recorded to 2 years following the drought period. A shorter follow-up period was investigated in sensitivity analyses. If a person had a gap of over a year between their last recorded visit date and the cohort/centre-specific administrative censoring date, then they were considered as lost-to-follow-up one year after their last recorded visit date.

Models additionally adjusted for sex at birth, and data recorded at follow-up start on age (16–25, 26–35, 36–45, 46–55, 56–65, ≥ 66), CD4 count cells/mm^3^ (0–99, 100–199, 200–349, 350–499, ≥ 500, missing), viral load in copies/mL (< 400, ≥ 400, missing), AIDS status (no AIDS, AIDS, unknown – using WHO staging), and the time since starting ART (< 6 months, 6 months to 1 year, 1–3 years, 3–6 years, 6–10 years, and ≥ 10 years). The models were also adjusted for a binary variable to indicate locations with a Normalized Difference Vegetation Index (NDVI) value ≥ 0.3. NDVI assesses whether a pixel contains live green vegetation and is both an indicator of plant health and land use (https://gisgeography.com/ndvi-normalized-difference-vegetation-index/). NDVI values were taken on 1st Jun 2016 (when analyses started). Low values indicate a place has less vegetation. These data were linked to the IeDEA-SA data using the longitude and latitude of the HIV centres. To capture the values for CD4 and viral loads at follow-up start, a window from 1st January 2015 to 31st May 2016 was used, taking the value closest to follow-up start. Models were run including PWH from all HIV centres, as well as stratifying by rural/urban status of each HIV centre.

### Sensitivity analyses

For analyses of each outcome, sensitivity analyses were performed removing one cohort at a time and limiting follow-up time at 1 year instead of 2 years. For the analysis of mortality, two additional sensitivity analyses were performed 1) including the Kheth’Impilo cohort that did not have dates of death available and using logistic regression rather than Cox proportional hazards models because of this lack of data on death dates, and 2) not including the vital registration linkage that was available only for the South African cohorts.

### Analysis of visitors per HIV centre

We used generalized estimating equation regression models (Negative binomial, with an identity link) to investigate whether relative rainfall impacted the number of PWH attending HIV centres between June 2014 and May 2016. HIV centres from participating cohorts were included if they had at least 20 unique visitors in June 2014 and were still open by May 2016. The variables included in the models were the rainfall change variable, the NDVI ≥ 0.3 variable, urban/rural region, the number of months since June 2014, and the cohort. Models were re-run stratifying by rural and urban region. There were no missing data for any covariates included in this analysis.

## Results

**Fig. 1:**
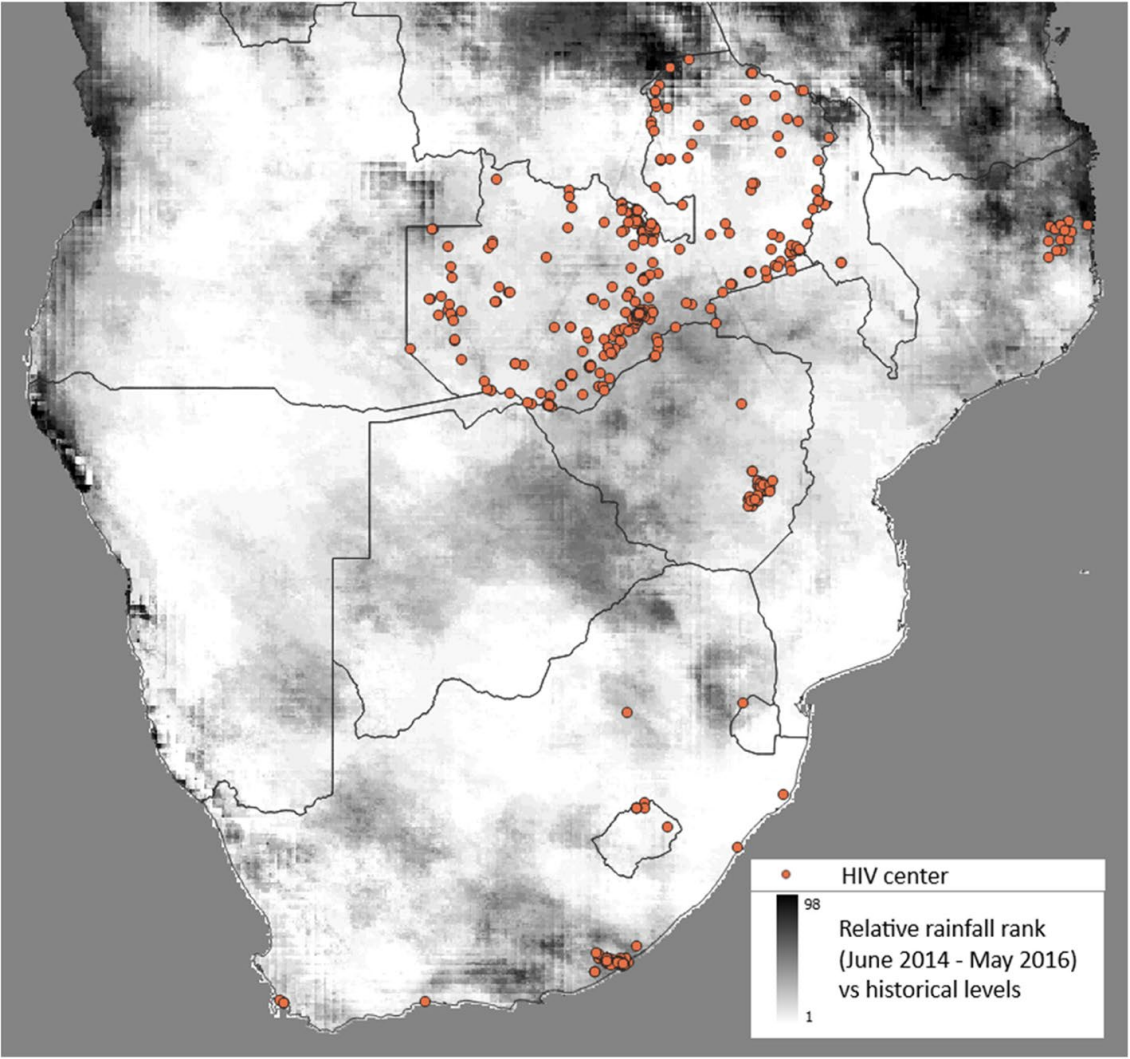
Locations of the HIV centres (red dots) and relative rainfall rank for June 2014 to May 2016 versus historical levels in an observational cohort study of the effect of rainfall on HIV outcomes in Southern Africa

Data on 843,289 PWH of all ages, on and off ART were available in the IeDEA-SA dataset, which spanned the period 1997–2021. Of these, 43,531 PWH were excluded due to having missing data on which HIV centre they had attended. Of the remainder, 270,708 adult PWH had started ART prior to 01/06/2016 and were still alive at this point and in follow-up in the year prior to this, so were eligible for inclusion in this study. These 270,708 PWH were included from 386 HIV centres (Fig. 1; Table 1), out of 393 in the overall dataset (5 centres had missing geocode coordinates and 2 were not operating at the start of follow-up). 181,722 (67.1%) of the 270,708 PWH were female and the median age was 39 years (interquartile range [IQR]: 32–46) (Table 2). The median empirical percentile of average rainfall for June 2014 to May 2016 versus historical values was 22 (interquartile range [IQR]: 4–31; range: 1–89), where 50 would indicate average rainfall. Of the 11 cohorts included, 6 and 3 were based entirely in urban and rural areas, respectively, with the other 2 containing a mix of urban and rural areas. For the 222,865 (82.3%) PWH that attended HIV centres in urban areas, the median rainfall percentile was 25 (IQR: 4–31; range: 1–54), whilst it was 10 (IQR: 7–25; range: 1–89) for the 47,843 (17.7%) PWH that attended HIV centres in rural areas. The number of PWH attending HIV centres in regions with an NDVI value ≥ 0.3 was 68,208 (25.2%). At the start of follow-up, the median length of time that the included persons had been on ART was 3.5 (IQR: 1.4, 6.3) years and 48,071 (17.8%) had experienced a prior AIDS event. Among the 154,091 (56.9%) PWH with data on CD4 cell count at follow-up start, the median value was 425 (IQR: 270–600) cells/mm^3^.

**Table 1:** Characteristics of each HIV cohort in an observational cohort study of the effect of rainfall on HIV outcomes in Southern Africa

Cohort	Country	Number of centres	Number of PWH	Rural	Urban	Median rainfall change rank (IQR)
CIDRZ	Zambia	241	121,247	18,766 (15.5%)	102,481 (84.5%)	25 (16–31)
Gugulethu Community Health Centre	South Africa	1	3131	0 (0.0%)	3131 (100.0%)	16 (16–16)
Hlabisa	South Africa	1	6331	6331 (100.0%)	0 (0.0%)	1 (1–1)
Khayelitsha	South Africa	4	27,076	0 (0.0%)	27,076 (100.0%)	4 (4–4)
Kheth’Impilo	South Africa	83	45,323	0 (0.0%)	45,323 (100.0%)	33 (28–39)
Lighthouse Trust	Malawi	2	28,947	0 (0.0%)	28,947 (100.0%)	4 (4–4)
Newlands Clinic	Zimbabwe	1	4391	0 (0.0%)	4391 (100.0%)	22 (22–22)
SMART Lesotho	Lesotho	6	2772	2428 (87.6%)	344 (12.4%)	7 (7–7)
SMART Mozambique	Mozambique	12	7016	7016 (100.0%)	0 (0.0%)	60 (51–63)
SMART Zimbabwe	Zimbabwe	34	13,302	13,302 (100.0%)	0 (0.0%)	10 (7–13)
Themba Lethu	South Africa	1	11,172	0 (0.0%)	11,172 (100.0%)	22 (22–22)
**Overall**		**386**	**270,708**	**47,843 (17.7%)**	**222,865 (82.3%)**	**22 (4–31)**

*IQR* Interquartile range. *PWH* Persons with HIV

**Table 2:** Time-updated characteristics at follow-up start (1st June 2016) of included PWH, stratified by rural or urban HIV centre location in an observational cohort study of the effect of rainfall on HIV outcomes in Southern Africa

Variable	Rural	Urban	Overall
Male	15,531 (32.5%)	73,455 (33.0%)	88,986 (32.9%)
Female	32,312 (67.5%)	149,410 (67.0%)	181,722 (67.1%)
16–25 years old	4462 (9.3%)	16,862 (7.6%)	21,324 (7.9%)
26–35 years old	12,593 (26.3%)	64,478 (28.9%)	77,071 (28.5%)
36–45 years old	16,048 (33.5%)	83,007 (37.3%)	99,055 (36.6%)
46–55 years old	9302 (19.4%)	42,006 (18.9%)	51,308 (19%)
56–65 years old	4193 (8.8%)	13,557 (6.1%)	17,750 (6.6%)
66 + years old	1245 (2.6%)	2955 (1.3%)	4200 (1.6%)
CD4 < 100 cells/mm^3^	1176 (2.5%)	8183 (3.7%)	9359 (3.5%)
CD4 100–199 cells/mm^3^	2234 (4.7%)	12,440 (5.6%)	14,674 (5.4%)
CD4 200–349 cells/mm^3^	5652 (11.8%)	27,941 (12.5%)	33,593 (12.4%)
CD4 350–499 cells/mm^3^	6555 (13.7%)	31,580 (14.2%)	38,135 (14.1%)
CD4 500 + cells/mm^3^	10,536 (22%)	47,743 (21.4%)	58,279 (21.5%)
CD4 cells/mm^3^ missing	21,690 (45.3%)	94,978 (42.6%)	116,668 (43.1%)
HIV-1 viral load < 400	4545 (9.5%)	87,247 (39.2%)	91,792 (33.9%)
HIV-1 viral load ≥ 400	629 (1.3%)	10,736 (4.8%)	11,365 (4.2%)
HIV-1 viral load missing	42,669 (89.2%)	124,882 (56.0%)	167,551 (61.9%)
< 6 months on ART	3972 (8.3%)	21,072 (9.5%)	25,044 (9.3%)
6–12 months on ART	4087 (8.5%)	20,673 (9.3%)	24,760 (9.2%)
1–2 years on ART	14,010 (29.3%)	58,078 (26.1%)	72,088 (26.6%)
3–5 years on ART	14,479 (30.3%)	60,350 (27.1%)	74,829 (27.6%)
6–9 years on ART	10,253 (21.4%)	48,764 (21.9%)	59,017 (21.8%)
10 + years on ART	1042 (2.2%)	13,928 (6.3%)	14,970 (5.5%)
No prior AIDS	43,210 (90.3%)	179,427 (80.5%)	222,637 (82.2%)
Prior AIDS	4633 (9.7%)	43,438 (19.5%)	48,071 (17.8%)
In region with NDVI < 0.3	18,302 (38.3%)	184,198 (82.7%)	202,500 (74.8%)
In region with NDVI ≥ 0.3	29,541 (61.8%)	38,667 (17.4%)	68,208 (25.2%)
**Overall**	**47,843 (100%)**	**222,865 (100%)**	**270,708 (100%)**

*NDVI* Normalised Difference Vegetation Index, with higher values indicating more vegetation, *ART* Antiretroviral Therapy

### Mortality

For the analyses examining mortality, 104,138 PWH were included from 9 cohorts, among whom there were 2,951 deaths (2.8%) in 327,414 person-years. In the overall analysis (Table 3; Supplementary Table 1), decreases in relative rainfall levels versus historical values were associated with higher mortality, adjusted hazard ratio (aHR) 1.18 (95% confidence interval [95%CI]: 1.07–1.32) per 10 percentile decrease in rainfall. In analyses of PWH in rural sites, these results persisted. For PWH in urban sites, there was no association between mortality and changes in rainfall due to a lack of within-cohort variation in rainfall for the subgroup included in the analysis. The association persisted across 12/13 sensitivity analyses (Supplementary Table 2), although the confidence interval was much wider when dropping the SMART Mozambique cohort, aHR 1.08 (95%CI: 0.78–1.49). The association was stronger when using 1-year follow-up after the drought period, rather than 2 years, aHR 1.36 (95%CI 1.14–1.61).

**Table 3:** Adjusted^a^ hazard ratios and odds ratios for each outcome measure per decrease of 10 percentiles in rainfall versus historical values in an observational cohort study of the effect of rainfall on HIV outcomes in Southern Africa

		**Per 10 percentile decrease in rainfall**
**Mortality**	**N PWH (N with outcome)**	**Adjusted hazard ratios (95% confidence interval)**
Overall	104,138 (2951)	1.18 (1.07–1.32)
Urban	75,061 (1899)	NA^b^
Rural	29,077 (1052)	1.18 (1.06–1.32)
**CD4 counts < 200 cells/mm3**	**N PWH (N with outcome)**	**Adjusted odds ratios (95% confidence interval)**
Overall	27,580 (3990)	0.94 (0.89–1.00)
Urban	23,154 (3167)	0.94 (0.88–1.01)
Rural	4426 (823)	0.99 (0.89–1.10)
**HIV-1 viral loads > 400 copies/mL**	**N PWH (N with outcome)**	**Adjusted odds ratios (95% confidence interval)**
Overall	82,860 (13,788)	1.05 (1.01–1.09)
Urban	77,519 (13,341)	1.05 (1.01–1.09)
Rural	4741 (447)	Did not converge
** ≥ 12-month gaps in care**	**N PWH (N with outcome)**	**Adjusted hazard ratios (95% confidence interval)**
Overall	270,708 (9426)	0.98 (0.94–1.01)
Urban	222,865 (8015)	0.96 (0.92–1.00)
Rural	47,843 (1411)	0.86 (0.80–0.93)

^a^Adjusted for cohort, male/female sex at birth, a binary variable to indicate locations with a Normalized Difference Vegetation Index (NDVI) value ≥ 0.3, and age, CD4 count cells/mm3, viral load copies/mL, AIDS status, and the time since starting ART time-updated at follow-up start

^b^Result due to there being no within-cohort variation in rainfall

*PWH* People with HIV

### CD4 counts < 200 cells/mm.^3^

When investigating CD4 counts < 200 cells/mm^3^ as the outcome, 27,580 PWH were included from 3 cohorts, among whom 3,990 (14.5%) had a CD4 count < 200 cells/mm^3^ recorded during follow-up (Table 3; Supplementary Table 3). In the overall analysis, there was weak evidence of a negative association between decreases in relative rainfall levels versus historical values and having a CD4 count < 200 cells/mm^3^, adjusted odds ratio (aOR) 0.94 (95%CI: 0.89–1.00) per 10% percentile decrease in the rainfall level. When stratifying by rural/urban location, the association was very similar in urban areas and was much attenuated in rural areas, aOR 0.99 (0.89–1.10). Results for 5/5 of the sensitivity analyses were similar to those in the main analysis (Supplementary Table 4).

### HIV-1 viral loads > 400 copies/mL

For the analysis with HIV-1 viral load > 400 copies/mL as the outcome, 6 cohorts were included containing 82,260 PWH, among whom 13,788 (16.8%) had viral loads > 400 copies/mL recorded during follow-up (Table 3; Supplementary Table 5). In the overall analysis, lower relative rainfall levels were positively associated with having a viral load > 400 copies/mL recorded, aOR 1.05 (95%CI: 1.01–1.09) per 10 percentile decrease in rainfall. This analysis could not be repeated in rural sites due to a lack of data on this outcome, but the results persisted for PWH in urban areas. In 7/8 of the sensitivity analyses, similar odds ratios and p-values were seen (Supplementary Table 6), but the results attenuated substantially when removing the Kheth’Impilo cohort, which is driving the overall association (there was no within-cohort variation in rainfall among the remaining cohorts).

###  ≥ 12-month gaps in care

All 11 cohorts and 270,708 PWH were included in the analyses of ≥ 12-month gaps in care (Table 3; Supplementary Table 7). There were 9,426 PWH who had a ≥ 12-month gap in 567,272 person-years. Overall, there was no evidence of an association between relative rainfall levels versus historical values and ≥ 12-month gaps in care, aHR 0.98 (95%CI: 0.94–1.01), per 10% decrease. However, when stratifying by urban and rural location, there was evidence of a protective association between lower relative rainfall and ≥ 12-month gaps in care in both urban and rural areas, aORs 0.96 (0.92–1.00) and 0.86 (0.80–0.93), respectively, per 10 percentile decrease in rainfall rank. In the 12 sensitivity analyses, the results remained consistent with the overall analysis (supplementary Table 8).

### Analysis of visitors per HIV centre

For the analysis of numbers of unique PWH visiting HIV centres per month, 187 HIV centres were included. The median number of unique PWH visiting each HIV centre per month increased from 196 in June 2014 to 220 in May 2016 (Supplementary Fig. 1). Each December saw a drop in urban areas. The numbers of visitors were higher in urban than in rural areas. Table 4 shows the associations of relative rainfall levels with monthly numbers of unique visitors per HIV centre. In fully adjusted analyses, HIV centres in areas with lower levels of rainfall than usual had lower numbers of PWH visiting them, with the number of unique visitors reducing by a factor of 0.80 (95%CI: 0.66–0.98) per 10% decrease in rainfall. There was a positive association between the number of visitors and the number of months since January 2014, i.e. the clinics became busier over time. In both the rural and urban regions, the point estimate for the association between relative rainfall levels and monthly clinic visitors was similar to the overall analysis, but confidence intervals were wider: numbers of unique visitors decreased by factors of 0.80 (0.53–1.19) and 0.83 (0.66–1.05) for rural and urban areas, respectively, per 10 percentile decrease in rainfall rank.

**Table 4:** Adjusted rate ratios for the numbers of unique visitors per HIV centre for each month between June 2014 and May 2016 in an observational cohort study of the effect of rainfall on HIV outcomes in Southern Africa

	**Rate ratio (95% confidence interval) of unique visitors to HIV centres**	
**Overall**	**Adjusted for cohort**	**Fully adjusted**^a^
Per relative 10 percentile decrease in rainfall^b^	0.77 (0.64–0.94)	0.80 (0.66–0.98)
Months since Jan 2014	1.04 (1.02–1.05)	1.03 (1.02–1.05)
NDVI ≥ 0.3	0.63 (0.37–1.08)	0.90 (0.50–1.61)
Urban region	1.75 (0.97–3.14)	1.43 (0.78–2.60)
**Rural region**	**Adjusted for cohort**	**Fully adjusted**^a^
Per relative 10 percentile decrease in rainfall^b^	0.81 (0.56–1.19)	0.80 (0.53–1.19)
Months since Jan 2014	0.98 (0.95–1.01)	0.99 (0.95–1.01)
NDVI ≥ 0.3	1.04 (0.53–2.02)	1.15 (0.57–2.34)
**Urban region**	**Adjusted for cohort**	**Fully adjusted**^a^
Per relative 10 percentile decrease in rainfall^b^	0.79 (0.64–0.98)	0.83 (0.66–1.05)
Months since Jan 2014	1.06 (1.04–1.08)	1.05 (1.04–1.07)
NDVI ≥ 0.3	0.35 (0.13–0.93)	0.50 (0.17–1.44)

*NDVI* Normalised Difference Vegetation Index

^a^Adjusted for the variables shown in table, as well as for cohort

^b^A rate ratio below 1 indicates that HIV centres with less rainfall than historically have fewer unique visitors

## Discussion

We found higher mortality among PWH on ART living in regions with lower rainfall than usual, both overall and when including just rural areas. We also found higher odds of unsuppressed viral loads among PWH on ART in regions with lower-than-normal rainfall, overall and in urban areas. However, we did not find convincing evidence of relationships between decreases in rainfall and recording low CD4 counts or ≥ 12-month gaps in care among PWH on ART. Additionally, we found that HIV centres in areas with less rainfall than usual had lower numbers of PWH visiting them. Overall, among PWH on ART we saw some evidence of negative HIV treatment outcomes in areas with lower rainfall than historically. However, the evidence was not consistent across outcomes or when stratifying by rural/urban location, and, therefore, the pathways between decreases in rainfall and HIV outcomes require further elucidation.

### Comparisons with other literature

Climate change is hypothesised to negatively impact on HIV treatment outcomes via increases in food insecurity and poverty [8], although evidence on this is limited. There have been various studies that have established that food insecurity, which is linked to poverty, can adversely impact on HIV treatment outcomes [22–24]. This includes systematic reviews on the relationships between food insecurity and adherence to ART [13], HIV-1 viral suppression [16], and CD4 count [15]. However, to our knowledge, this is the first study to look at the effect of drought or rainfall levels on ART outcomes including mortality. Otherwise, previous cross-sectional studies of weather and HIV in sub-Saharan Africa have found associations between local rainfall shocks and heightened HIV prevalence [25], as well as between drought and increased HIV prevalence and riskier sexual behaviour in young women in rural areas [26]. A recent study in South Africa found that adherence and retention in care decreased during years of drought [14]. Another study found evidence that unusually heavy rainfall was associated with a higher HIV burden across 21 sub-Saharan African countries [27]. Other analyses have also found that droughts increased transactional sex among women employed in agriculture in Malawi, as well as HIV prevalence among both men and women [28]. Finally, a study across 10 sub-Saharan African countries found that droughts were associated with lower odds of HIV testing and higher odds of condomless sex [29]. That much of this literature found associations between droughts and HIV-related outcomes among women living in rural areas show both the gender and urban/rural divides in the pathways linking drought and HIV. Studies suggest that the impacts of climate on HIV are seen particularly among women because of greater dependence on transactional sex [30], and that the effects are concentrated in rural areas, due to changes in climate having very direct impacts on the livelihoods and behaviours of farmers, whilst any affects in urban areas would likely be less direct, potentially occurring through drought affecting the general economy [14]. Other literature has looked at the effects of drought in sub-Saharan Africa on other health outcomes that, like HIV, would be more indirectly affected by climate. This includes studies suggesting that childhood vaccination levels are reduced in the presence of drought, potentially due to drought creating extra barriers to clinic access [31].

### Strengths and limitations

A strength of this analysis is the use of a very large, longitudinal cohort of PWH on ART spanning multiple Southern African countries combined with a measure of change in rainfall. This has allowed us to analyse outcomes that occurred after these exposures were measured, removing the difficulties of interpreting observed associations in cross-sectional data. However, there are several limitations. Although in each analysis we adjusted for cohort, the associations seen between lower rainfall and HIV treatment outcomes could instead be capturing other differences between regions. The cohorts capture different populations and some of the associations seen could be reflecting cross-cohort and cross-country differences in unmeasured confounders, such as types of HIV care provider, treatment practices, income, and food insecurity. Missing data on potential confounders is a common issue with routinely collected data. Furthermore, there is differential reporting and recording of variables between cohorts, including of CD4 cell counts and viral loads, and in the analyses of mortality, linkage to vital registration was only possible for some of the South African cohorts. In particular, few cohorts collected robust data on CD4 count monitoring due to a transition to viral load monitoring around this time [19]. Therefore, the analysis of CD4 counts only contained data from 3 cohorts. The inconsistency of the results across the various outcome measures (CD4 counts, viral loads, gaps in care, and mortality) could be due to the differential recording of these variables, particularly in the South African cohorts where there was linkage to death registry data, so this will have been recorded more fully than the HIV biomarkers. To account for these various limitations, we performed many sensitivity analyses, including removing the vital registration linkage for the South African cohorts, and investigating how leaving out each cohort from each analysis affected the results. Additionally, several cohorts only had one or two centres, meaning that there was no within-cohort variation in rainfall. This meant that the cohorts with more centres were more influential in the analyses and further limited the number of cohorts included and variation in rainfall that was captured when stratifying analyses by rural and urban areas. Potential mechanisms between rainfall and HIV epidemiology are less clear in areas where there is little subsistence farming, particularly urban areas (which often draw their water from dams located elsewhere), so, any associations in analyses specific to urban areas should be interpreted with caution. We also do not know the timespan in which rainfall levels will affect treatment outcomes – this requires further research. However, we investigated using a shorter 1-year follow-up duration in sensitivity analyses and found similar results for low CD4 counts, unsuppressed viral loads, and 12-month gaps in care, and a stronger association between decreased rainfall and increased mortality than when using 2 years of follow-up. For the analysis of unique visitor numbers, this could have been affected by lengthening ART dispensing periods over time, although this would likely have been happening across all HIV cohorts and centres. As data were only available on the locations of the HIV centres, rather than on place of residence, we had to assume that their place of residence had comparable weather to their HIV centre. When analysing mortality, we were only able to look at all-cause mortality rather than cause-specific mortality, so were unable to understand whether the increased mortality in regions with less rainfall than usual was AIDS-related or due to other causes.

## Conclusion

Our results add to the evidence that changes in rainfall can be associated with the epidemiology of HIV in high HIV prevalence areas such as Southern Africa through changing behaviours [28, 29]. Previous research has highlighted that food insecurity and income are key links in the chain between climate change and HIV [12]. Additional research is required using longitudinal cohort data where information on these links can be accounted for to further our understanding. Improvements in HIV care could mitigate the potential future negative effects of reduced rainfall on HIV treatment outcomes, particularly in rural areas. However, the frequency and severity of droughts are projected to increase in Southern Africa [7], which could blunt the impact of improvements in HIV care on treatment outcomes, hindering progress towards UNAIDS’ goals of controlling the HIV epidemic by 2030 [32]. Additionally, climate change could cause increased conflict over land and resources, with such conflicts also likely to negatively impact on the HIV care cascade [33]. Our results regarding the effect of drought on HIV treatment outcomes, also have implications for the monitoring and treatment of other chronic conditions that may worsen in drought situations, although reviews have noted a surprising lack of research into this topic [34, 35]. Whilst human-driven climate change is a global issue that will require global efforts and investment to deal with [1], environmental and HIV interventions could be considered in high HIV burden areas that are likely to experience droughts. Localised interventions in Southern Africa that could mitigate the effects of climate change on HIV outcomes could include the use of newly developed long-lasting antiretrovirals that do not need to be taken daily [36], multi-month dispensing [37], microcredit interventions to reduce reliance on subsistence agriculture [38], reducing the travel costs and time associated with obtaining ART [39, 40], and building early warning systems to help health systems anticipate extreme weather conditions [41].

### Supplementary Information

**Additional file 1: Supplementary table 1. **Full results of the analysis with mortality as the outcome.** Supplementary table 2. **Sensitivity analysis results for the analysis with mortality as the outcome.** Supplementary table 3. **Full results for the analysis with CD4 counts<200 cells/mm3 as the outcome.** Supplementary table 4. **Sensitivity analysis results for the analysis with CD4 counts<200 cells/mm3 as the outcome.** Supplementary table 5. **Full results for the analysis with viral loads≥400 copies/mL as the outcome.** Supplementary table 6. **Sensitivity analysis results for the analysis with viral loads≥400 copies/mL as the outcome. **Supplementary table 7. **Full results for the analysis with 12-month gaps in care as the outcome. **Supplementary table 8. **Sensitivity analyses results for the analysis with 12-month gaps in care as the outcome.** Supplementary table 9.** Strengthening the Reporting of Observational studies in Epidemiology (STROBE) checklist. **Supplementary Figure 1. **Median unique PWH visiting each HIV centre per month.

## Data Availability

The data file designed by the World Food Programme's Vulnerability Analysis and Mapping Geospatial Analysis Team that compares historical rainfall patterns with rainfall between June 2014 to May 2016 is available on request—please contact the corresponding author for access as the datasets are very large. The HIV cohort data are not publicly available. Please contact IeDEA-SA for further information (https://www.iedea-sa.org/).
