# Supplementary material for: Associations of inter-annual rainfall decreases with subsequent HIV outcomes for persons with HIV on antiretroviral therapy in Southern Africa: a collaborative analysis of cohort studies
Source: BMC Infect Dis. 2023 Dec 19;23:889. doi: 10.1186/s12879-023-08902-9 (PMC10731689; doi:10.1186/s12879-023-08902-9)
Supplement: Supplementary file 1 — Additional file 1: Supplementary table 1. Full results of the analysis with mortality as the outcome. Supplementary table 2. Sensitivity analysis results for the analysis with mortality as the outcome. Supplementary table 3. Full results for the analysis with CD4 counts<200 cells/mm3 as the outcome. Supplementary table 4. Sensitivity analysis results for the analysis with CD4 counts<200 cells/mm3 as the outcome. Supplementary table 5. Full results for the analysis with viral loads≥400 copies/mL as the outcome. Supplementary table 6. Sensitivity analysis results for the analysis with viral loads≥400 copies/mL as the outcome. Supplementary table 7. Full results for the analysis with 12-month gaps in care as the outcome. Supplementary table 8. Sensitivity analyses results for the analysis with 12-month gaps in care as the outcome. Supplementary table 9. Strengthening the Reporting of Observational studies in Epidemiology (STROBE) checklist. Supplementary Figure 1. Median unique PWH visiting each HIV centre per month. [file 12879_2023_8902_MOESM1_ESM.docx]

**Associations of inter-annual rainfall decreases with subsequent HIV outcomes for persons with HIV on antiretroviral therapy in Southern Africa: a collaborative analysis of cohort studies**

**SUPPLEMENTARY MATERIALS**

#

# Climate context from late 2014 to mid-2016

# *This section has been taken from *“Food insecurity and the risk of HIV acquisition: findings from population-based surveys in six sub-Saharan African countries (2016-2017)”* by Low et al.^1^ and amended, with the permission of the authors.

Analysis carried out by the World Food Programme (WFP) on rainfall and temperature patterns in the past 40 years for several countries in the African continent show that while there are marked temperature increases across the region, the case is less clear cut for rainfall.

The key rainfall feature for agricultural production and consequently rural food production and food insecurity is the inter-annual (year-on-year) variability in rainfall. In the long run, rainfall variability is a major determinant of livelihoods in the semi-arid tropics as beyond a certain value, purely agriculture-based livelihoods become unfeasible, and households switch progressively to livestock-based livelihoods. These fluctuations subject households to the twin hazards of drought and flood. It is a long-term driver of chronic food insecurity as large and unpredictable year-on-year fluctuations in rainfall amounts prevent households from diversifying the crops they plant and lead them to become more risk averse and conservative in terms of their production strategies. In general, the magnitude of inter-annual variations is much larger than any changes arising from a possible long-term trend.

Another mode of variation in seasonal rainfall that may be present is associated with multi-year periods of drier or wetter than average conditions with inter-annual variability super-imposed on these lower frequency cycles. While changes in mean and extreme rainfall have been the object of intense study, rainfall variability has received much less attention. Recent studies indicate that in response to global warming, rainfall variability in tropical areas is expected to increase more than mean precipitation due to greater increase in rainfall extremes^2^.

From late 2014 to mid-2016 one of the longest and most intense El Niño events on record developed. For Southern Africa in particular, it led to two consecutive droughts, the second of which had very intense impacts on regional food insecurity. Consecutive droughts have compounding effects on food insecurity – the first drought, besides depleting national and regional stocks and direct impacts on households, enhances their vulnerability due erosion of household savings and sale of productive of assets. This enhances the impacts of the second drought through severe reductions in staple food availability and extreme market prices. This was followed by two La Niña events in 2016-2017 and 2017-2018, which nevertheless led to drier than average conditions in East Africa and wetter conditions in Southern Africa.

**MORTALITY**

**Supplementary table 1:** Full results of the analysis with mortality as the outcome

| **Variable** | **Hazard ratio (95% confidence interval)** |
| --- | --- |
| Per 10 percentile decrease in rainfall | 1.18 (1.07, 1.32) |
| NDVI≥0.3 | 1.03 (0.83, 1.28) |
| Prior AIDS | 1.39 (1.26, 1.53) |
| Female | 0.74 (0.68, 0.79) |
| Age 16-25 years | 1 |
| Age 26-35 years | 1.00 (0.84, 1.19) |
| Age 36-45 years | 1.12 (0.95, 1.33) |
| Age 46-55 years | 1.45 (1.21, 1.73) |
| Age 56-65 years | 2.53 (2.10, 3.04) |
| Age 66+ years | 4.36 (3.49, 5.44) |
| CD4 <100 cells/mm^3^ | 1 |
| CD4 100-199 cells/mm^3^ | 0.55 (0.47, 0.64) |
| CD4 200-349 cells/mm^3^ | 0.29 (0.25, 0.34) |
| CD4 350-499 cells/mm^3^ | 0.21 (0.18, 0.25) |
| CD4 500+ cells/mm^3^ | 0.15 (0.13, 0.17) |
| CD4 cells/mm^3^ missing | 0.21 (0.18, 0.24) |
| HIV-1 viral load <400 | 1 |
| HIV-1 viral load ≥400 | 2.28 (1.98, 2.61) |
| HIV-1 viral load missing | 1.14 (1.02, 1.28) |
| <6 months on ART | 1 |
| 6-12 months on ART | 0.88 (0.74, 1.05) |
| 1-2 years on ART | 0.98 (0.85, 1.13) |
| 3-5 years on ART | 0.99 (0.86, 1.14) |
| 6-9 years on ART | 0.99 (0.85, 1.15) |
| 10+ years on ART | 0.97 (0.80, 1.18) |

NDVI: Normalised difference vegetation index. ART: Antiretroviral therapy.

**Supplementary table 2:** Sensitivity analysis results for the analysis with mortality as the outcome

| **Analysis** | **Per 10 percentile decrease in rainfall** |
| --- | --- |
|  | **Hazard ratio (95% confidence interval)** |
| Include Kheth'Impilo | 1.10 (1.04-1.17) |
| Drop Gugulethu | 1.18 (1.07-1.32) |
| Drop Hlabisa | 1.18 (1.06-1.31) |
| Drop Khayelitsha | 1.17 (1.06-1.30) |
| Drop Lighthouse | 1.19 (1.07-1.32) |
| Drop Newlands | 1.18 (1.07-1.31) |
| Drop Smartles | 1.19 (1.07-1.32) |
| Drop Smartmoz | 1.08 (0.78-1.49) |
| Drop Smartzim | 1.21 (1.08-1.35) |
| Drop Thembalethu | 1.19 (1.07-1.32) |
| Without death registry data | 1.18 (1.06-1.31) |
| Restricting follow-up to 1^st^ Jun 2017 | 1.36 (1.14-1.61) |

**CD4 COUNTS<200 CELLS/MM3**

**Supplementary table 3:** Full results for the analysis with CD4 counts<200 cells/mm3 as the outcome.

| **Variable** | **Odds ratio (95% confidence interval)** |
| --- | --- |
| Per 10 percentile decrease in rainfall | 0.94 (0.89, 1.00) |
| NDVI≥0.3 | 1.33 (1.08, 1.65) |
| Prior AIDS | 1.28 (1.18, 1.40) |
| Female | 0.67 (0.62, 0.72) |
| Age 16-25 years | 1 |
| Age 26-35 years | 1.08 (0.93, 1.26) |
| Age 36-45 years | 1.17 (1.01, 1.35) |
| Age 46-55 years | 1.11 (0.95, 1.30) |
| Age 56-65 years | 1.20 (0.99, 1.47) |
| Age 66+ years | 0.97 (0.68, 1.38) |
| CD4 <100 cells/mm^3^ | 1 |
| CD4 100-199 cells/mm^3^ | 0.39 (0.34, 0.44) |
| CD4 200-349 cells/mm^3^ | 0.08 (0.07, 0.09) |
| CD4 350-499 cells/mm^3^ | 0.02 (0.02, 0.03) |
| CD4 500+ cells/mm^3^ | 0.01 (0.01, 0.02) |
| CD4 cells/mm^3^ missing | 0.10 (0.09, 0.11) |
| HIV-1 viral load <400 | 1 |
| HIV-1 viral load ≥400 | 4.52 (4.04, 5.06) |
| HIV-1 viral load missing | 1.83 (1.63, 2.04) |
| <6 months on ART | 1 |
| 6-12 months on ART | 1.11 (0.96, 1.28) |
| 1-2 years on ART | 1.80 (1.58, 2.05) |
| 3-5 years on ART | 1.74 (1.51, 2.01) |
| 6-9 years on ART | 1.67 (1.43, 1.96) |
| 10+ years on ART | 1.38 (1.06, 1.79) |
| Time since starting ART (years) | 1.00 (1.00, 1.00) |
| KHETHIMPILO cohort | 1 |
| NEWLANDS cohort | 1.16 (0.89, 1.50) |
| SMARTMOZ cohort | 0.96 (0.74, 1.24) |

NDVI: Normalised difference vegetation index. ART: Antiretroviral therapy.

**Supplementary table 4:** Sensitivity analysis results for the analysis with CD4 counts<200 cells/mm3 as the outcome.

| **Analysis** | **Per 10 percentile decrease in rainfall** |
| --- | --- |
|  | **Odds ratio (95% confidence interval)** |
| Drop KHETHIMPILO | 0.98 (0.88-1.09) |
| Drop NEWLANDS | 0.94 (0.89-1.00) |
| Drop SMARTMOZ | 0.94 (0.88-1.01) |
| Only KHETHIMPILO | 0.94 (0.88-1.01) |
| Restricting follow-up to 1^st^ Jun 2017 | 0.94 (0.89-1.00) |

**VIRAL LOADS≥400 COPIES/ML**

**Supplementary table 5:** Full results for the analysis with viral loads≥400 copies/mL as the outcome

| **Variable** | **Odds ratio (95% confidence interval)** |
| --- | --- |
| Per 10 percentile decrease in rainfall | 1.05 (1.01, 1.09) |
| NDVI≥0.3 | 0.66 (0.38, 1.15) |
| Prior AIDS | 1.20 (1.15, 1.26) |
| Female | 0.80 (0.77, 0.84) |
| Age 16-25 years | 1 |
| Age 26-35 years | 0.70 (0.64, 0.76) |
| Age 36-45 years | 0.58 (0.53, 0.63) |
| Age 46-55 years | 0.51 (0.46, 0.55) |
| Age 56-65 years | 0.45 (0.41, 0.51) |
| Age 66+ years | 0.46 (0.38, 0.56) |
| CD4 <100 cells/mm^3^ | 1 |
| CD4 100-199 cells/mm^3^ | 0.84 (0.77, 0.93) |
| CD4 200-349 cells/mm^3^ | 0.58 (0.53, 0.63) |
| CD4 350-499 cells/mm^3^ | 0.42 (0.38, 0.46) |
| CD4 500+ cells/mm^3^ | 0.33 (0.30, 0.36) |
| CD4 cells/mm^3^ missing | 0.47 (0.43, 0.51) |
| HIV-1 viral load <400 | 1 |
| HIV-1 viral load ≥400 | 9.32 (8.84, 9.82) |
| HIV-1 viral load missing | 2.08 (1.97, 2.20) |
| <6 months on ART | 1 |
| 6-12 months on ART | 1.32 (1.20, 1.44) |
| 1-2 years on ART | 1.89 (1.74, 2.06) |
| 3-5 years on ART | 1.93 (1.77, 2.10) |
| 6-9 years on ART | 2.04 (1.86, 2.23) |
| 10+ years on ART | 2.11 (1.89, 2.37) |
| Time since starting ART (years) | 1.00 (1.00, 1.00) |
| GUGULETHU cohort | 1 |
| HLABISA cohort | 1.92 (1.07, 3.45) |
| KHAYELITSHA cohort | 0.82 (0.69, 0.96) |
| KHETHIMPILO cohort | 3.70 (3.15, 4.35) |
| NEWLANDS cohort | 1.37 (0.76, 2.47) |
| THEMBALETHU cohort | 6.16 (5.26, 7.22) |

NDVI: Normalised difference vegetation index. ART: Antiretroviral therapy.

**Supplementary table 6:** Sensitivity analysis results for the analysis with viral loads≥400 copies/mL as the outcome

| **Analysis** | **Per 10 percentile decrease in rainfall** |
| --- | --- |
|  | **Odds ratio (95% confidence interval)** |
| Drop GUGULETHU | 1.05 (1.01-1.09) |
| Drop HLABISA | 1.05 (1.01-1.09) |
| Drop KHAYELITSHA | 1.05 (1.01-1.09) |
| Drop KHETHIMPILO | 0.04 (0.03-0.06)* |
| Drop NEWLANDS | 1.05 (1.01-1.09) |
| Drop THEMBALETHU | 1.05 (1.01-1.09) |
| Only KHETHIMPILO | 1.05 (1.02-1.09) |
| Restricting follow-up to 1^st^ Jun 2017 | 1.05 (1.00-1.10) |

*This result is due to there being no within-cohort variation in rainfall when Kheth’Impilo is removed.

**12-MONTH GAPS IN CARE**

**Supplementary table 7:** Full results for the analysis with 12-month gaps in care as the outcome

| **Variable** | **Hazard ratio (95% confidence interval)** |
| --- | --- |
| Per 10 percentile decrease in rainfall | 1.05 (1.01, 1.09) |
| NDVI≥0.3 | 0.66 (0.38, 1.15) |
| Prior AIDS | 1.20 (1.15, 1.26) |
| Female | 0.80 (0.77, 0.84) |
| Age 16-25 years | 1 |
| Age 26-35 years | 0.70 (0.64, 0.76) |
| Age 36-45 years | 0.58 (0.53, 0.63) |
| Age 46-55 years | 0.51 (0.46, 0.55) |
| Age 56-65 years | 0.45 (0.41, 0.51) |
| Age 66+ years | 0.46 (0.38, 0.56) |
| CD4 <100 cells/mm^3^ | 1 |
| CD4 100-199 cells/mm^3^ | 0.84 (0.77, 0.93) |
| CD4 200-349 cells/mm^3^ | 0.58 (0.53, 0.63) |
| CD4 350-499 cells/mm^3^ | 0.42 (0.38, 0.46) |
| CD4 500+ cells/mm^3^ | 0.33 (0.30, 0.36) |
| CD4 cells/mm^3^ missing | 0.47 (0.43, 0.51) |
| HIV-1 viral load <400 | 1 |
| HIV-1 viral load ≥400 | 9.32 (8.84, 9.82) |
| HIV-1 viral load missing | 2.08 (1.97, 2.20) |
| <6 months on ART | 1 |
| 6-12 months on ART | 1.32 (1.20, 1.44) |
| 1-2 years on ART | 1.89 (1.74, 2.06) |
| 3-5 years on ART | 1.93 (1.77, 2.10) |
| 6-9 years on ART | 2.04 (1.86, 2.23) |
| 10+ years on ART | 2.11 (1.89, 2.37) |

NDVI: Normalised difference vegetation index. ART: Antiretroviral therapy.

**Supplementary table 8:** Sensitivity analyses results for the analysis with 12-month gaps in care as the outcome

| **Analysis** | **Per 10 percentile decrease in rainfall** |
| --- | --- |
|  | **Hazard ratio (95% confidence interval)** |
| Drop CIDRZ | 0.96 (0.92-1.00) |
| Drop GUGULETHU | 0.98 (0.94-1.01) |
| Drop HLABISA | 0.98 (0.94-1.01) |
| Drop KHAYELITSHA | 0.97 (0.94-1.01) |
| Drop KHETHIMPILO | 0.99 (0.93-1.04) |
| Drop LIGHTHOUSE | 0.98 (0.95-1.01) |
| Drop NEWLANDS | 0.98 (0.94-1.01) |
| Drop SMARTLES | 0.98 (0.94-1.01) |
| Drop SMARTMOZ | 0.97 (0.93-1.00) |
| Drop SMARTZIM | 0.97 (0.94-1.01) |
| Drop THEMABLETHU | 0.98 (0.94-1.01) |
| Restricting follow-up to 1^st^ Jun 2017 | 0.99 (0.94-1.04) |

**Supplementary Figure 1:** Median unique PWH visiting each HIV centre per month.

**Supplementary table 9:** STrengthening the Reporting of OBservational studies in Epidemiology (STROBE) checklist.

|  | Item No | Recommendation | Page |
| --- | --- | --- | --- |
| **Title and abstract** | 1 | (*a*) Indicate the study’s design with a commonly used term in the title or the abstract | 1 (title) |
|  |  | (*b*) Provide in the abstract an informative and balanced summary of what was done and what was found | 3 (abstract) |
| Introduction | | |  |
| Background/rationale | 2 | Explain the scientific background and rationale for the investigation being reported | 4 (paragraphs 1-3) |
| Objectives | 3 | State specific objectives, including any prespecified hypotheses | 4 (paragraph 3) |
| Methods | | |  |
| Study design | 4 | Present key elements of study design early in the paper | 5 (Cohort data) |
| Setting | 5 | Describe the setting, locations, and relevant dates, including periods of recruitment, exposure, follow-up, and data collection | 5 (Cohort data), Table 1, Figure 1 |
| Participants | 6 | (*a*) Give the eligibility criteria, and the sources and methods of selection of participants | 5, 7 (Individual-level inclusion criteria, Analysis of visitors per HIV centre) |
| Variables | 7 | Clearly define all outcomes, exposures, predictors, potential confounders, and effect modifiers. Give diagnostic criteria, if applicable | 5-7 (Rainfall data, Individual-level analyses, Analysis of visitors per HIV centre), Figure 1 |
| Data sources/ measurement | 8 | For each variable of interest, give sources of data and details of methods of assessment (measurement). Describe comparability of assessment methods if there is more than one group | 5-7 (Rainfall data, Individual-level analyses, Analysis of visitors per HIV centre) |
| Bias | 9 | Describe any efforts to address potential sources of bias | 6 (Individual-level analyses) |
| Study size | 10 | Explain how the study size was arrived at | 5 (Individual-level inclusion criteria) |
| Quantitative variables | 11 | Explain how quantitative variables were handled in the analyses. If applicable, describe which groupings were chosen and why | 6 (Individual-level analyses) |
| Statistical methods | 12 | (*a*) Describe all statistical methods, including those used to control for confounding | 6 (Individual-level analyses) |
|  |  | (*b*) Describe any methods used to examine subgroups and interactions | 6-7 (Individual-level analyses, Analysis of visitors per HIV centre) |
|  |  | (*c*) Explain how missing data were addressed | 5 (Individual-level inclusion criteria) |
|  |  | (*d*) If applicable, describe analytical methods taking account of sampling strategy | Not applicable |
|  |  | (*e*) Describe any sensitivity analyses | 6 (Individual-level analyses) |
| Results | | |  |
| Participants | 13 | (a) Report numbers of individuals at each stage of study—eg numbers potentially eligible, examined for eligibility, confirmed eligible, included in the study, completing follow-up, and analysed | 8-9 (Results) |
|  |  | (b) Give reasons for non-participation at each stage | 5 (Individual-level inclusion criteria) |
|  |  | (c) Consider use of a flow diagram | Not applicable |
| Descriptive data | 14 | (a) Give characteristics of study participants (eg demographic, clinical, social) and information on exposures and potential confounders | 8 (Results), Table 2 |
|  |  | (b) Indicate number of participants with missing data for each variable of interest | 8-9 (Results), Table 2 |
| Outcome data | 15 | Report numbers of outcome events or summary measures | 8-9 (Results) |
| Main results | 16 | (*a*) Give unadjusted estimates and, if applicable, confounder-adjusted estimates and their precision (eg, 95% confidence interval). Make clear which confounders were adjusted for and why they were included | 8-9 (Results), Table 3, Table 4. |
|  |  | (*b*) Report category boundaries when continuous variables were categorized | 6 (Individual-level analyses), Table 2 |
|  |  | (*c*) If relevant, consider translating estimates of relative risk into absolute risk for a meaningful time period | Not applicable |
| Other analyses | 17 | Report other analyses done—eg analyses of subgroups and interactions, and sensitivity analyses | Supplementary tables 2, 4, 6, and 8 |
| Discussion | | |  |
| Key results | 18 | Summarise key results with reference to study objectives | 10 (Discussion) |
| Limitations | 19 | Discuss limitations of the study, taking into account sources of potential bias or imprecision. Discuss both direction and magnitude of any potential bias | 10 (Strengths and limitations) |
| Interpretation | 20 | Give a cautious overall interpretation of results considering objectives, limitations, multiplicity of analyses, results from similar studies, and other relevant evidence | 11 (Conclusions) |
| Generalisability | 21 | Discuss the generalisability (external validity) of the study results | 10 (Strengths and limitations) |
| Other information | | |  |
| Funding | 22 | Give the source of funding and the role of the funders for the present study and, if applicable, for the original study on which the present article is based | 12 (Funding) |

**REFERENCES**

1. Low A, Gummerson E, Schwitters A, et al. Food insecurity and the risk of HIV acquisition: findings from population-based surveys in six sub-Saharan African countries (2016-2017). *Bmj Open* 2022; **12**(7).

2. Pendergrass AG, Knutti R, Lehner F, Deser C, Sanderson BM. Precipitation variability increases in a warmer climate. *Sci Rep* 2017; **7**(1): 17966.
